# Mechanics and Energetics of Excavation by Burrowing Wolf Spiders, *Geolycosa* spp.

**DOI:** 10.1673/031.011.0122

**Published:** 2011-02-24

**Authors:** Robert B. Suter, Gail E. Stratton, Patricia R. Miller

**Affiliations:** ^1^ Department of Biology, Vassar College, Poughkeepsie, New York USA; ^2^ Department of Biology, University of Mississippi, Oxford, Mississippi USA; ^3^ Department of Biology, Northwestern Mississippi Community College, Senatobia, Mississippi USA

**Keywords:** abrasive wear, fossorial, physiological cost

## Abstract

Burrowing wolf spiders, *Geolycosa* sp. (Araneae:Lycosidae), excavate vertical burrows and inhabit them throughout their lives or, in the case of males, until they mature and wander in search of mates. Three species: *G. fatifera* Kurata, *G. missouriensis* Banks, and *G. rogersi* Wallace were studied to understand how and at what expense the burrowing is accomplished. Normal and high-speed videography coupled with scanning electron microscopy revealed (a) that the convex surfaces of the two fangs, together, constitute the digging tool, (b) that boluses of soil are transported to the burrow entrance on the anterior surfaces of the chelicerae held there by the pedipalps, and (c) that each bolus is either incorporated into the growing turret or flung away, propelled by the forelegs. To elucidate the energetics of burrow construction, burrow volumes were calculated and then the costs associated with dislodging, elevating, and throwing the known volumes of soil were measured. A typical *Geolycosa* burrow, at a volume of 23.6 ± 2.0 ml and a depth of 13.2 ± 0.7 cm, required the removal of 918 boluses each weighing about 34 mg. The aggregate dislodging cost was close to 1.9 Joules in sand/sandy loam and 5.6 J in clayey subsoil, the work against gravity necessary to raise all of the boluses to the surface was about 0.13 J, and the aggregate cost of flinging the boluses was close to 0.014 J. Assuming that the ratio of external work to metabolic cost of external work is between 0.20 and 0.25 in spiders, the real cost of burrow construction would be between 8 J and 29 J, depending primarily on soil type. This is a small but not negligible cost when placed in the context of reproductive effort: a single *Geolycosa* egg, dozens to hundreds of which are produced in a clutch, contains about 10 J.

## Introduction

Many species of wolf spiders (Araneae, Lycosidae) excavate cavities in the ground and use them for retreats during various phases of their lives (Wallace 1942; unpublished data [GES]). Some, mainly those in the genus *Geolycosa*, but also a few elsewhere in the family (e.g. *Hogna carolinensis*) are obligate burrowers, constructing approximately vertical burrows that can be 15–30 body lengths in depth (Wallace 1942; Carrel 2003 and references therein).

**Figure 1.**
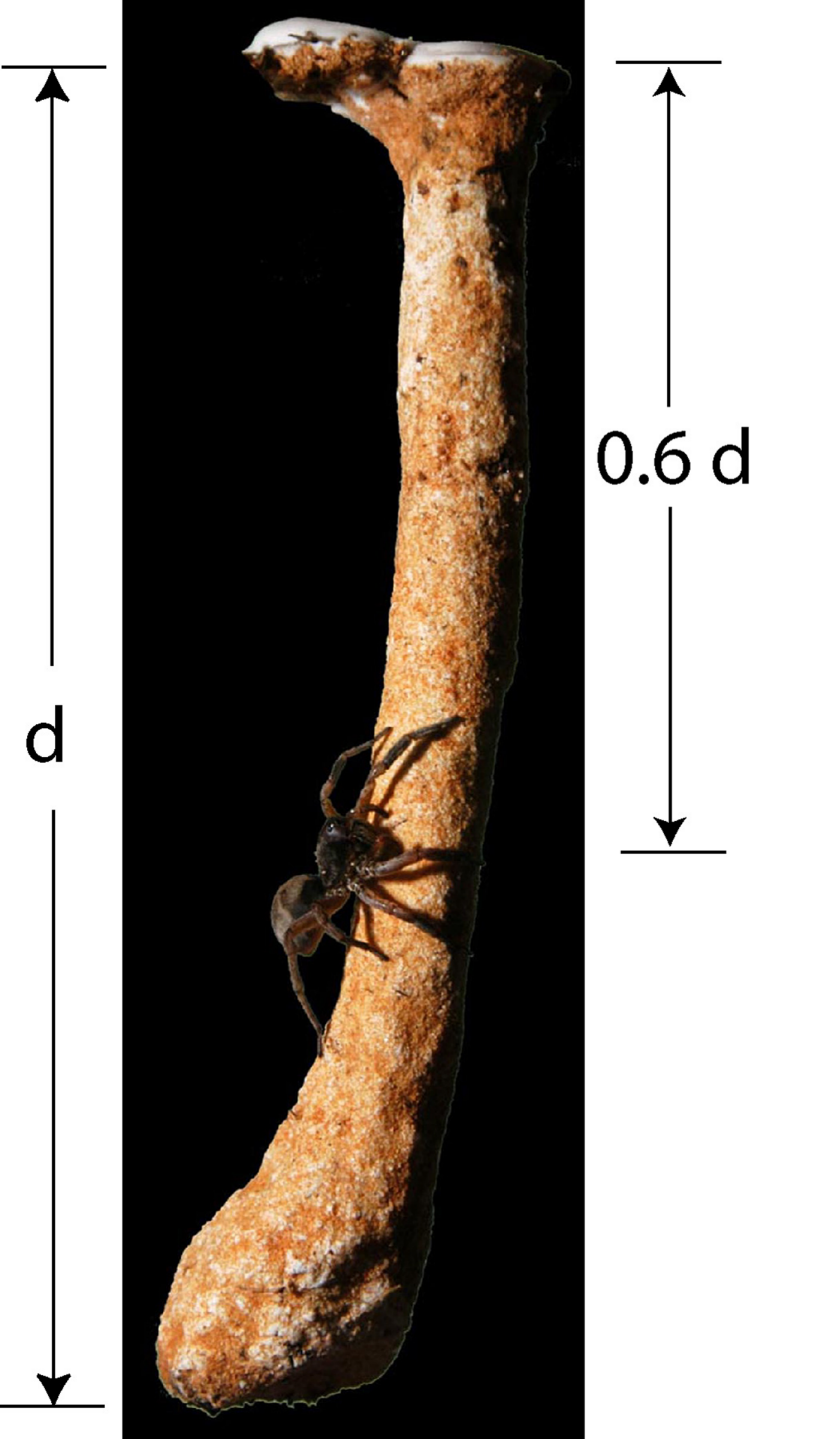
Female *Geolycosa missouriensis* on the plaster cast of its burrow. If the burrow were uniform in diameter from top to bottom (at depth d) half of the material extracted during burrow construction would come from below a depth of 0.5 d. Because the burrow is wider at the bottom, that halfway depth is at about 0.6 d. High quality figures are available online.

Among *Geolycosa* (Figure 1), burrows are excavated even by the very young and throughout the spiders' lives burrow site fidelity is high except among adult males (Wallace 1942; McCrone 1963; McQueen 1983; Miller 1989; but see also Richardson 1990; Marshall 1995). Moreover, these wolf spiders are tenacious inhabitants of their digs, rarely moving more than a few centimeters from the burrow entrance, retreating into the burrow at the slightest suggestion of danger, often remaining in the burrow during experimental flooding (personal observations, RBS), and resisting exposure even when tugged to the surface by a thread-tethered mealworm (personal observations, RBS); but burrow invasions by ants elicit rapid burrow abandonment (Marshall 1995). This tenacity, the site-fidelity, and the depth of the typical burrow, suggest that the fossorial life style is, for *Geolycosa*, nearly priceless.

**Figure 2.**
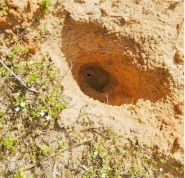
Burrow of a female *G. missouriensis* at the bottom of a hole dug by an armadillo. The hole was about 15 cm deep — had the spider's original burrow been shallower, the spider probably would not have survived. High quality figures are available online.

As with any adaptation, it is worth asking what the costs and benefits are. This is a core question about the architectural products of animals (e.g. Hansell 2000), just as it is about the timing of dispersal of hatchlings (e.g. Bonte et al. 2007) or about particular foraging techniques (e.g. concerning silk structure and function, Vollrath 1999). Among the species of wolf spiders that burrow (*Geolycosa* and others), the presumed or demonstrated benefits of inhabiting a burrow are at least these:

Predator avoidance/defense (Personal Observations, Figure 2; but see also Gwynne 1979; Conley 1985)
Desiccation reduction (Humphreys 1975)
Behavioral thermoregulation (Humphreys 1978a)

And at the same time, there are presumed or demonstrated costs:

Excavation energetics (this paper)
Constrained terrestrial dispersal (Miller and Miller 1991)
Reduced prey availability (Santana et al. 1990, using data from Humphreys 1975)
Reduced detectability by potential mates (Miller and Miller 1986)

In this study, the energetic costs of burrow construction were studied and the techniques used by *Geolycosa* during excavation were elucidated. The techniques and costs are closely linked not only in the physiology of the spiders, but also in the methods of analysis - the loosening of soil from the walls of the growing burrow, the transport of the packets (boluses) of soil to the surface, and the ejection of the boluses from the mouth of the burrow are behaviorally and energetically distinct phases of the process of excavation.

## Materials and Methods

### Spiders

Three species: *Geolycosa fatifera* (Kurata 1939), *G. missouriensis* (Banks 1895), and *G. rogersi* Wallace 1942 all observed *in situ* or captured at sites in Mississippi, USA were the subjects of this study. In the laboratory, where temperature varied between 20° C and 25° C and humidity was unregulated, those that had been captured were briefly held in 5 × 8.5 cm (142-ml) plastic vials before being transferred to test environments (described below) or killed and preserved in 95% ethyl alcohol. Ultimately, all were killed and preserved; voucher specimens have been deposited in the Mississippi Entomological Museum.

### Burrow dimensions and contents

In the field, burrows were filled with either Plaster of Paris (CaSO4∼0.5H2O) or leadenriched solder; these materials were allowed to solidify and the casts were extracted. Depth and diameter were determined using a ruler and calipers, and volume was found via fluid displacement in a graduated cylinder.

Both in the field and in the laboratory, the boluses of sand/sandy loam that spiders had ejected from the mouths of their burrows during excavation were collected. For each of 9 burrows (4 field, 5 lab), the pooled mass of 10 oven-dried boluses was measured and divided by 10 to arrive at an average dry mass/bolus. To know the wet mass of these boluses, freshly collected sand/sandy loam was collected, weighed, and then oven-dried to constant mass; the resulting water loss data allowed back-calculation from dry mass of pellets to wet mass of pellets. These data and the volumes of burrows allowed calculation of the approximate number of boluses a spider would have to collect, form, carry to the surface, and eject to complete excavation of a burrow of known volume.

### Burrowing behaviors

In the laboratory, a spider whose digging behavior was to be studied was released into a circular arena (diameter: 12 cm) containing 4– 5 cm of lightly compacted soil. To stimulate burrowing behavior (Miller 1984), a hemispheric depression in the soil surface was made approximately 1 cm in diameter and 0.5 cm deep at the edge of the arena where the spider, in its initial exploration of the environment, usually found it quickly and settled into it. The behaviors of multiple spiders were monitored simultaneously using iSight (Apple Inc.) color or lens board monochrome video cameras controlled by SecuritySpy® video surveillance software (bensoftware.com). Each camera was focused upon one hemispheric depression in the sand with the control software set to record periods of motion, but to ignore periods when the focal spider was inactive or out of the camera's view. The resulting videos were scrutinized at a variety of slowed playback speeds, but mostly at 30 frames per second.

The spiders' motions in casting away the materials they had excavated in their burrows were far too rapid to analyze using video captured at 30 frames per second. To study these motions, each spider was placed into a circular arena (diameter: 12 cm) containing lightly dampened sand/sandy loam collected at the site in Holmes County State Park (Mississippi) where most of the *G. missouriensis* specimens were captured. The soil was approximately 20 cm deep, again with a depression in the surface to foster burrowing at a predictable location. Once a spider's burrowing had progressed to a depth of several cm, its behavior at the burrow entrance was videotaped both in color at 30 frames per second and in monochrome at 500 frames per second (MotionScope S series, Redlake Imaging Corporation). The highspeed video was analyzed at 15 frames per second (about a 33-fold slowing) and frame by frame. Motions of parts of the spider and of the material it was ejecting were analyzed with the help of image analysis software (*NIH Image* and *ImageJ*, public domain software from the National Institutes of Health).

### Wear analysis

The fangs of spiders, like the wear-resistant parts of other arthropods, contain high levels both of heavy metals and of halogens (Schofield 2005) contributing to their hardness. Despite these chemical inclusions, the fangs still show wear and should show more wear in species that habitually excavate burrows than in species in which the fangs are used only in penetrating softer materials like insect cuticles. This hypothesis was tested using light microscopy to compare visually the wear on the fangs of *Geolycosa* sp. with wear on those of *Rabidosa rabida* (Walckenaer, 1837), a wolf spider of approximately the same size, but with much less inclination to construct burrows (Brady and McKinley 1994). For both, specimens that had just molted were selected so that not only well-used fangs (borne on the exuviae), but also pristine fangs (on the newly preserved whole spiders) were available for study.

In a second set of observations, designed to elucidate more directly the behaviors used in digging, spiders were placed on lightly compacted sand/sandy loam 1.5 cm deep. About half of the soil was supported from below by an additional two cm of the loam, while the other half was supported by a rigid foam block wrapped for about 70% of its area by sandpaper (silicon carbide abrasive, mean particle diameter, 190 µm, 3M Wetordry™ 431Q), with the abrasive surface of the sandpaper facing upward. As a spider dug downward in the latter situation it encountered first soil, then a single layer of paper tissue (Kimwipes®EX-L) that served to mark where digging had occurred, and finally the abrasive barrier or the foam block. The fangs of spiders that were subjects in this experiment, some of which burrowed to the sandpaper and others of which burrowed only in the loam or to the foam block, were later inspected under SEM for conspicuous wear.

### Scanning electron microscopy

Specimens to be viewed with the SEM were preserved in 100% EtOH, then freeze-dried, and finally sputter-coated with gold and palladium in a ratio of 80:20.

### Energetics analyses

To develop a complete estimate of the energy expenditures required to excavate a burrow of average depth and volume, one needs independent measures of the costs of dislodging the substrate, of transporting the substrate to the surface, and of dispersing the substrate once it is outside the deepening burrow.

**Figure 3.**
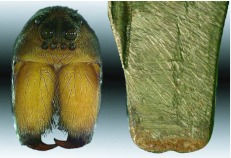
The face of a mature female *G. missouriensis* (left) and the brass pseudo-fangs that were fabricated for use in determining (Figure 4) how much work was needed to dislodge substrate during burrow construction. High quality figures are available online.

**Figure 4.**
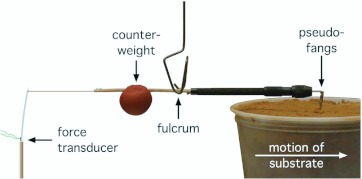
The device used to measure the work required in dislodging sandy loam or clayey subsoil during burrow excavation. The pseudo-fangs (Figure 3.) press down onto the substrate with a force determined by the location of the counterweight relative to the fulcrum. As the substrate is moved to the right, the horizontal force causing a scraping motion is measured by the force transducer and associated electronics. High quality figures are available online.

Dislodging soil requires some kind of scraping, presumably with an anatomical structure that is resistant to wear. The fangs are likely candidates for this role, particularly in the absence of a conspicuously distorted or prominent piece of anatomy, like the marginal teeth on the chelicerae of some trapdoor spiders (Araneae, Antrodiaetidae; Coyle 1975). To emulate the fangs of *Geolycosa* for experimental purposes, a piece of brass was machined so that its bottom edge, the part that would come into contact with soil, was of the same dimensions and shape as the distal parts of the spider's chelicerae with the fangs partially flexed (Figure 3). Those pseudofangs were, in effect, pulled across the formerly subterranean surfaces of two different kinds of soil: one being the clayey subsoil (derived from the underlying chalk formations) from which *G. fatifera* had been captured, and the other being the fine sand/sandy loam in which *G. missouriensis* had been found. (Note that these soils represent two ends of the range of soils in which *Geolycosa* are found in Mississippi.) In these tests, the brass pseudo-fangs were held nearly perpendicular to the soil surface where they exerted a constant downward force that could be adjusted by changing the position of a counterweight (Figure 4). Prior to each test, the surface of the soil was gently vacuumed without direct contact to remove all loose particles; and after each test, the vacuum procedure was repeated with all loose particles being collected for subsequent weighing.

## Results

### Burrowing behavior and fang wear

**Figure 5.**
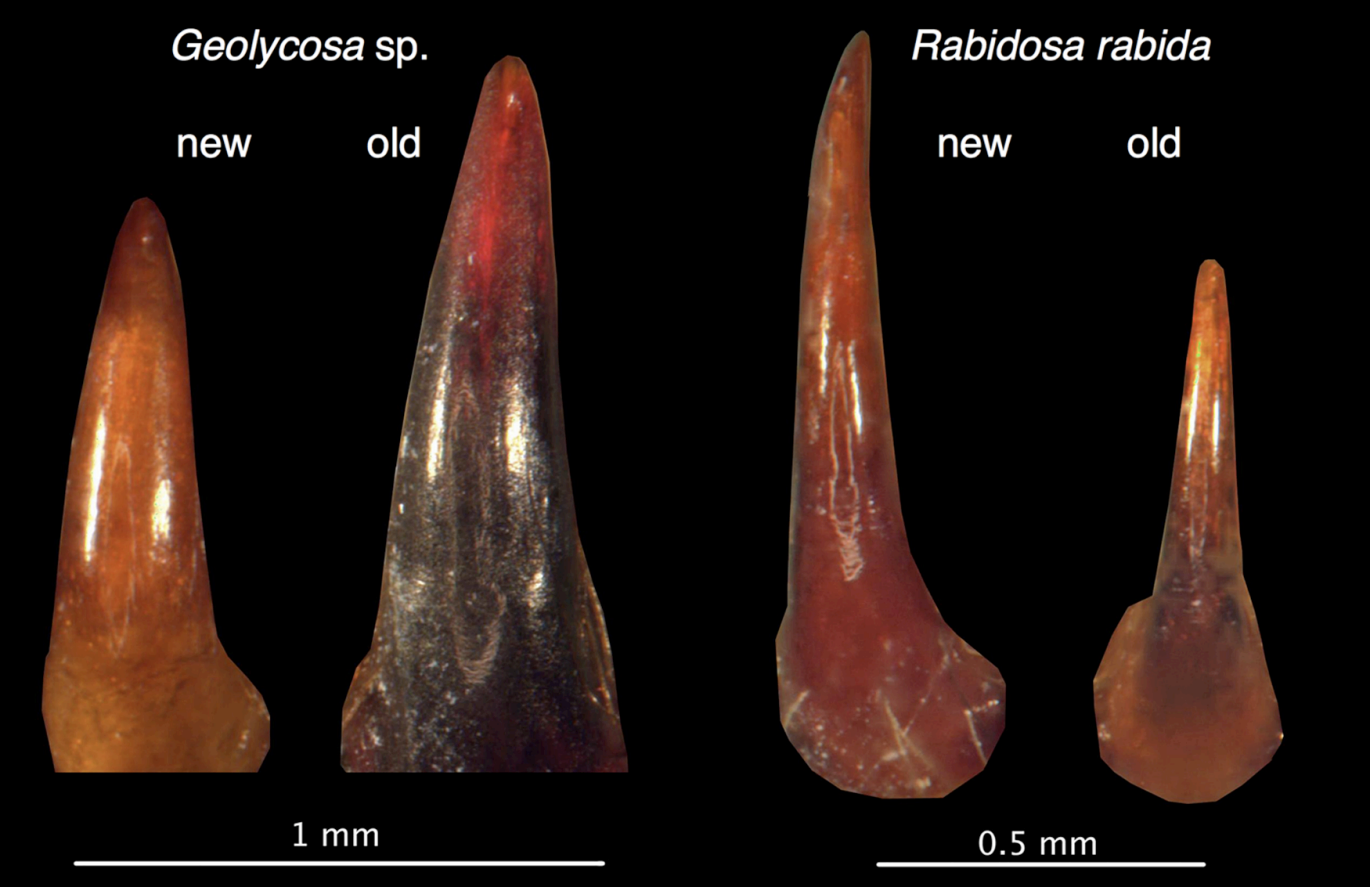
Two pairs of fangs, from *Geolycosa* (left) and *Rabidosa* (right). In each pair, the old fang was taken from the shed skin of the spider and the new fang was taken from the spider itself. The difference in wear in the burrowing spider's pair is noticeable, whereas there is very little difference in wear between the old and new fangs of the non-burrowing *R. rabida*. (Because the two *Geolycosa* fangs are strongly curved and were photographed at somewhat different angles, the new one appears, erroneously, to be smaller than the old one). High quality figures are available online.

**Figure 6.**
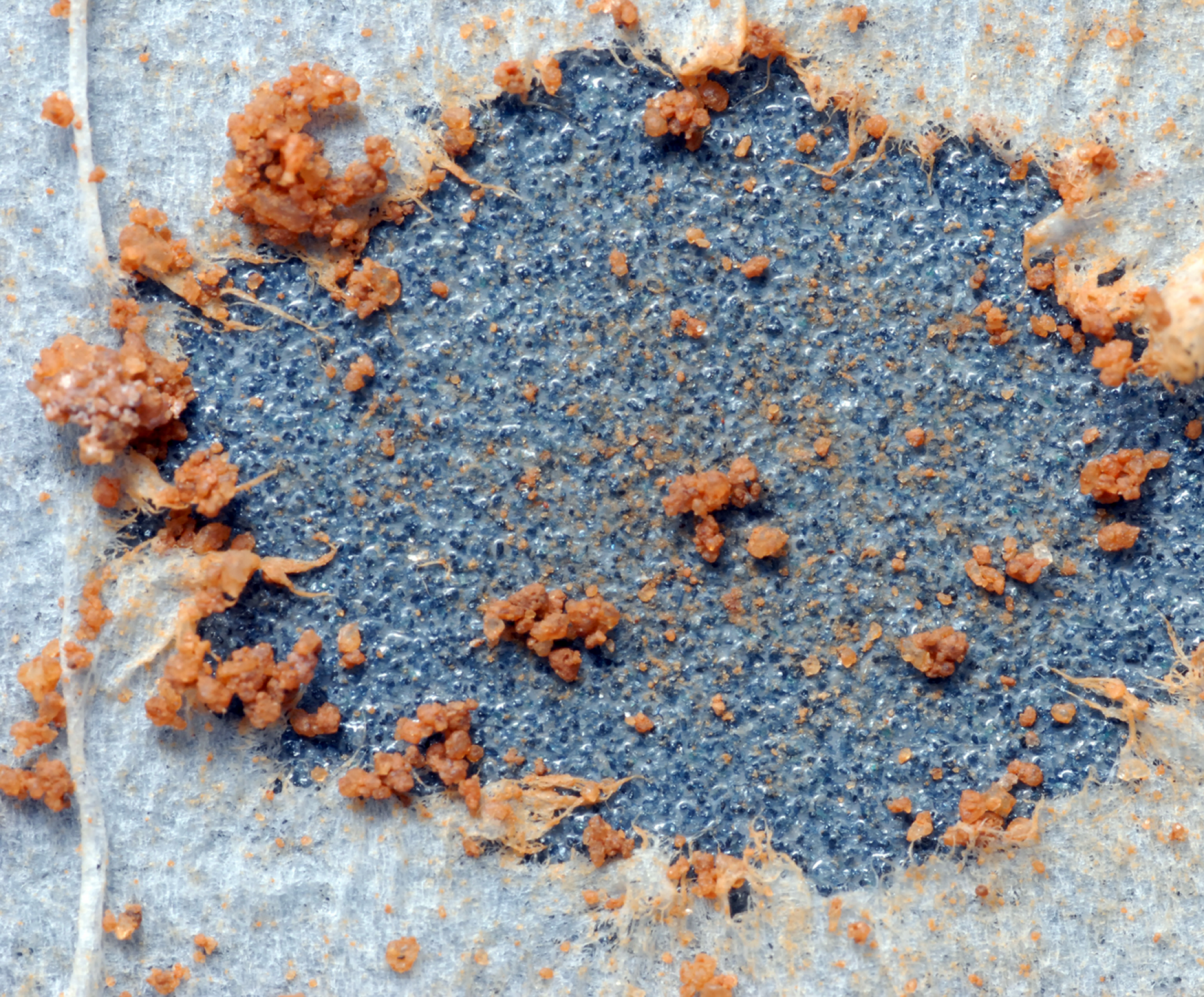
Top view of the site where a burrowing wolf spider dug through 1.5 cm of sandy loam and a layer of tissue paper and then attempted, unsuccessfully, to dig through foam-backed sandpaper. Attempts such as this caused scrapes on the fangs, visible under scanning electron microscopy (Figure 7). High quality figures are available online.

**Figure 7.**
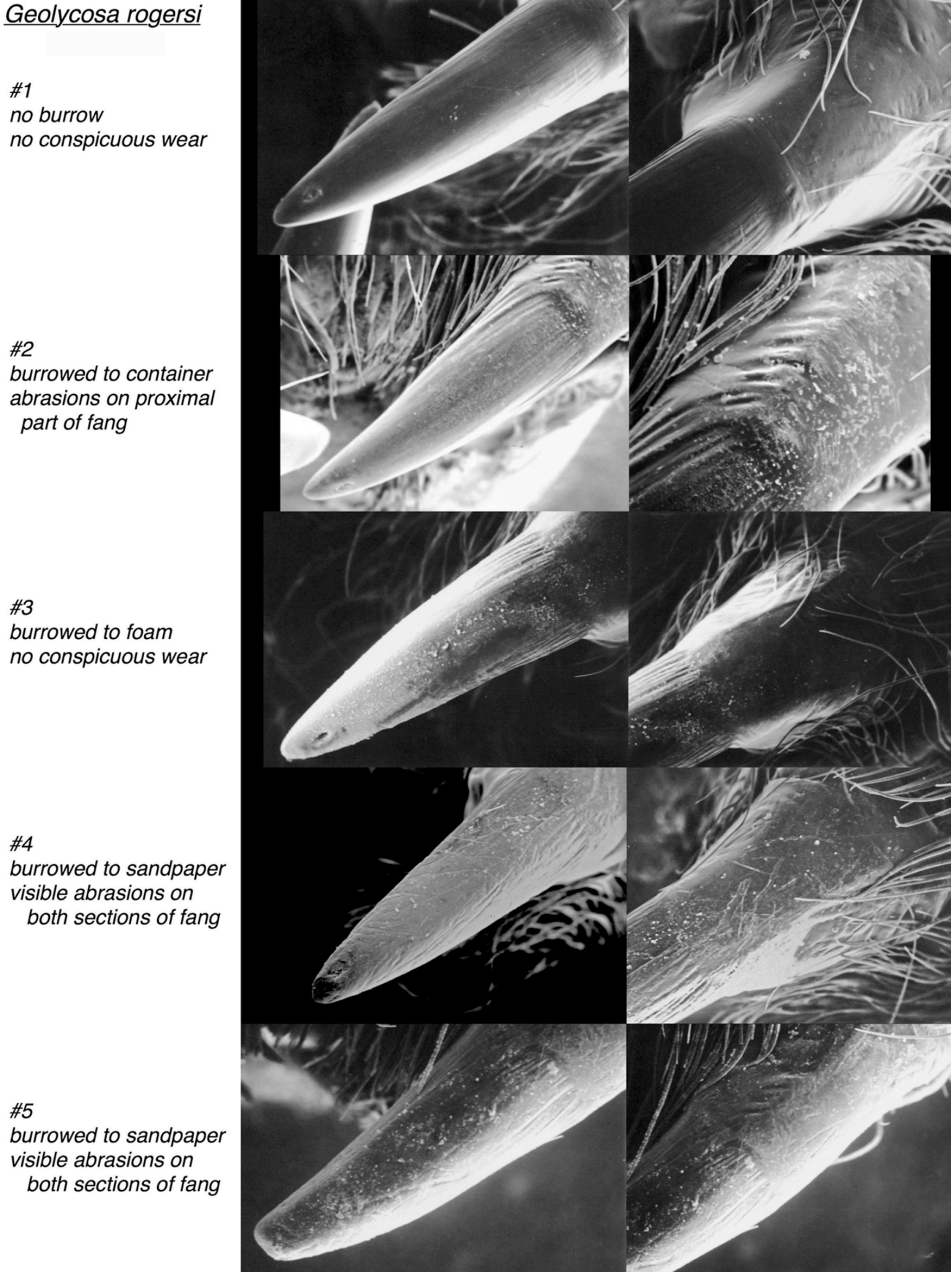
Scanning electron micrographs of the distal two thirds (left) and the proximal one third (right) of the fangs of five burrowing spiders that had the opportunity to dig through sandy loam and onto a sandpaper-covered block (Figure 6). The two spiders that attempted to penetrate the sandpaper, #4 and #5, had the most abrasions on their fangs. High quality figures are available online.

**Figure 8.**
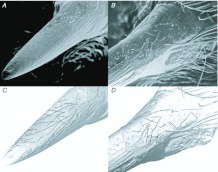
(A) and (B): Scanning electron micrographs of one fang (#4 from Figure 7) with the visible scratches emphasized (C and D) by tracing them on negative views of the same images. High quality figures are available online.

Video sequences captured at 30 frames per second showed that during excavation a *Geolycosa* appeared to use its legs to brace itself while using its fangs and perhaps also the lower fifth of its chelicerae to dislodge soil from the walls and floor of its deepening burrow (Video 1). Reinforcing the conclusion that the fangs are the primary tools used to loosen soil are two further results. First, visible wear on the fangs of *Rabidosa rabida*, a species of wolf spider in which burrowing is uncommon, was minor when compared to the complete absence of wear on newly exposed fangs whereas visible wear on the fangs of burrowing wolf spiders (*Geolycosa* spp.) was conspicuous when the same comparison was made (Figure 5 shows a typical comparison). Second, the fangs of burrowing wolf spiders that attempted to dig through a layer of sandpaper (Figure 6) were substantially more damaged than were the fangs of spiders that did not make such an attempt (Figure 7). The orientations of individual scratches (Figure 8; from fang #4 in Figure 7) indicated that much of the abrasive work done by sandpaper on the fang occurred while the fang was moving obliquely across the sandpaper; that is, many of the scratches were neither perpendicular nor parallel to the long axis of the fang (Figure 8, C and D).

**Video 1.**
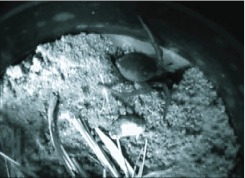
A burrowing wolf spider in the initial stages of excavation at the edge of a laboratory enclosure. Features of note are (1) the spider's use of its legs to brace itself while digging with the fangs and chelicerae, (2) the rapid motions of the first and second pairs of legs while forming the loosened soil into a pellet or bolus, and (3) the ejection of the bolus (30 frames per second). Click to view video. Download Video

**Figure 9.**
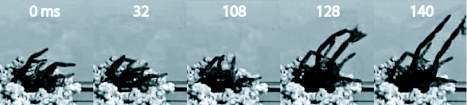
Five frames from Video 2, captured at 500 frames per second, showing steps in the ejection of a bolus from the mouth of a burrow. At t=0 ms, the spider's forelegs were being pulled back toward the face as the spider switched from locomotion (climbing up to the burrow entrance) to ejection. At 32 ms, the forelegs were partially cocked and the pedipalps were clearly visible holding the pale-toned bolus on top of the chelicerae. By 108 ms, the forelegs were fully cocked and positioned just behind the bolus. At 128 ms, the spider had accelerated the bolus to nearly its peak velocity, after which the bolus was free and its velocity and trajectory could be measured. High quality figures are available online.

In terms of process, the spider loosened substrate, formed it into a pellet or bolus, and carried it to the surface on the anterior face of its chelicerae held there by the pedipalps (Figure 9). At the surface it either added the bolus to those already silked into place as part of the turret, or it lofted the bolus onto the surrounding ground. In throwing the bolus the spider flexed its forelegs, placing the anterior surface of each against the back of the bolus, and then rapidly extended the forelegs accelerating the bolus away from the burrow entrance (Figure 9, Video 2). In both the field and the lab, the ejected boluses landed 6–50 cm from the burrow entrance, sometimes roughly evenly distributed in all directions and sometimes concentrated in a fan-shaped debris field spanning an arc of about 60 degrees.

### Energetic analysis

**Figure 10.**
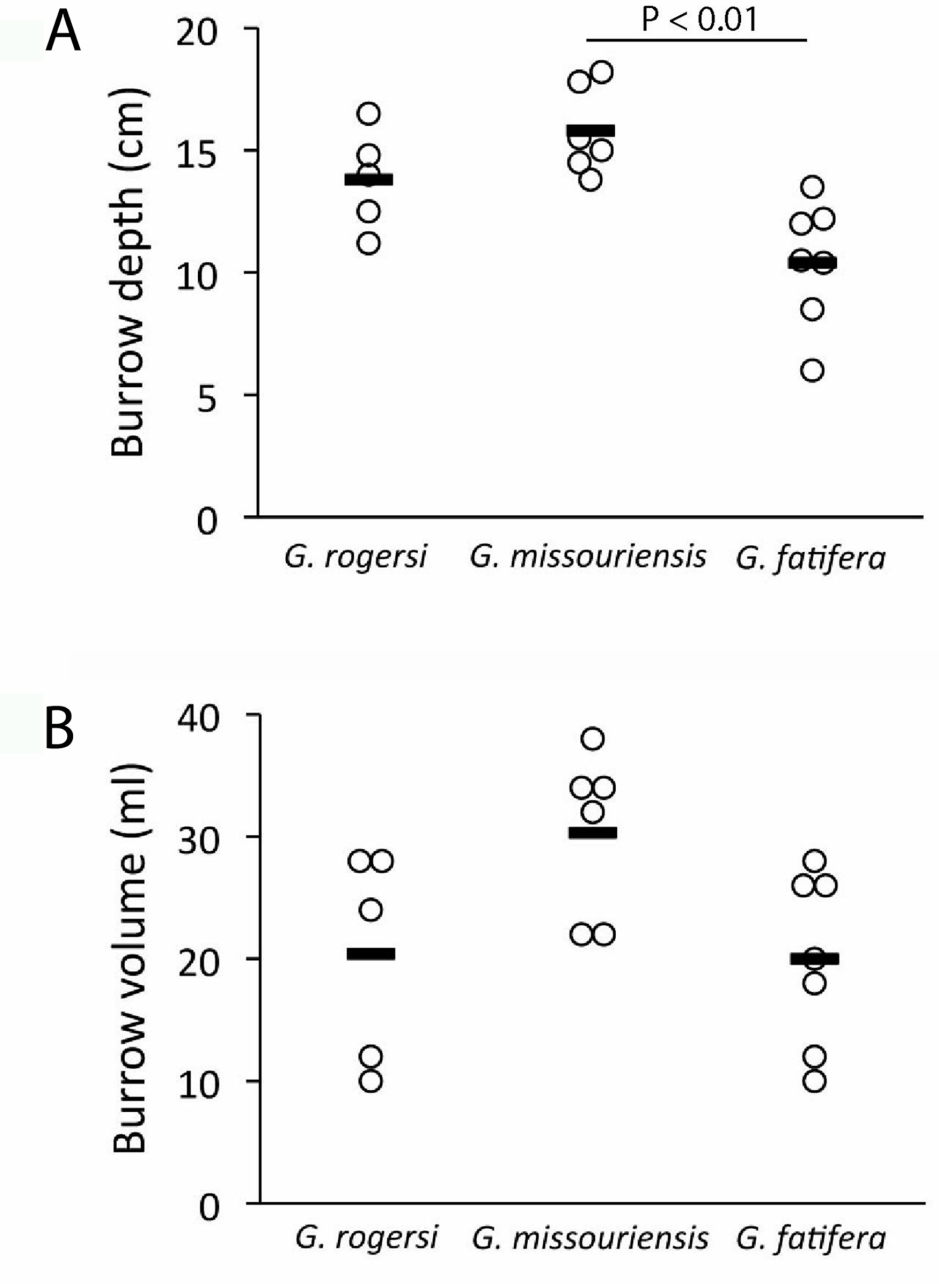
(A) Burrow depths varied significantly among the three species, with pair-wise testing indicating that the burrows of *G. missouriensis* were significantly deeper than those of *G. fatifera*. (B) There were no significant pair-wise differences among the burrows with respect to volume. High quality figures are available online.

**Burrow volumes and number of boluses.** The burrow depths and volumes of 5 *G. rogersi*, 6 *G. missouriensis*, and 7 *G. fatifera* were measured. A one-way ANOVA on burrow depths (Figure 10a) showed that there were significant differences among the species (F_2,15_ = 10.05, P = 0.0017). Tukey's Multiple Comparison Test revealed only one significant pair-wise comparison, between *G*. *missouriensis* and *G. fatifera* (q=6.244, P < 0.01). Despite this significant difference, for subsequent calculations the mean of the pooled depths, 13.16 ± 0.74 cm (mean ± S.E., here and below), was used. Although a oneway ANOVA on burrow volume (Figure 10b) showed that there was a marginally significant difference among the species (F_2,15_ = 3.702, P = 0.0493), Tukey's Multiple Comparison Test revealed no significant pair-wise differences at α=0.05. Consequently, for convenience in later calculations, the mean value for the pooled volumes, 23.56 ± 2.02 ml, was used.

Substrate density for the sand/sandy loam where *G. missouriensis* was found was 1.370 g/ml and for the clayey subsoil where *G*. *fatifera* was found was 1.301 g/ml; the average of these, 1.34 g/ml, was taken as the working value for substrate density. The pellets ejected from field- and laboratoryexcavated burrows had masses of 0.0344 ± 0.0070 g. The product of burrow volume and substrate density gave a value of 31.57 g for the mass of substrate ejected during the construction of an average burrow. Dividing this by the average mass of an ejected pellet revealed that the number of pellets a spider had to form, carry to the surface, and eject during burrow excavation was 917.7.

**Video 2.**
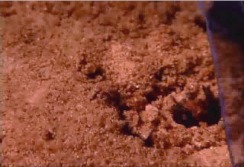
Several views of bolus ejection from the entrance to a deepening burrow. First, shown in color and at normal speed are two iterations of bolus ejection. Second, shown in black-and-white and at about 1/17 of normal speed is an example of bolus ejection as seen from a distance, allowing visualization of the trajectory of the bolus. And third are two close-ups of bolus ejection, the first at about 1/17 of normal speed and the second at about 1/50 of normal speed. In the latter clip, the actions of the forelegs in propelling the bolus are readily visible (see Figure 9 for further details). Click to view video. Download Video

**Figure 11.**
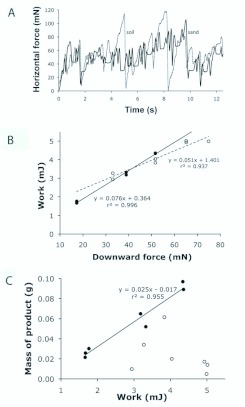
Work required to dislodge substrate (sandy loam or clayey subsoil), measured using the apparatus shown in Figure 4. (A) The time course of two experiments indicates the kind of data from which work values (force × distance) were derived. (B) The work done increased with the downward force on the pseudofangs, and the slopes of these relationships were different depending on whether the substrate was sandy loam (solid line) or clayey subsoil (dashed line). (C) The mass of dislodged substrate increased with increasing work when the substrate was sandy loam (solid line), but not when the substrate was clayey subsoil. High quality figures are available online.

**Dislodging soil from burrow walls.** It takes work (force applied over distance) to do the scraping required to dislodge soil from the walls of a growing burrow. Figure 11a shows two examples of the raw data (force vs. time) used as the basis for calculations of the work needed to dislodge soil. Modeling of the scraping process on fine sand/sandy loam and clayey soil showed that increasing downward pressure of the pseudo-fangs onto the surface during scraping caused a linear increase in the work required to pull the pseudo-fangs 0.07 m across the surface (Figure 11b); slopes of the lines were both significantly different from zero (sandy loam: F_1,5_ = 1234, P < 0.0001; clayey soil: F_1,5_ = 74.77, P = 0.0003) and from each other (F_1,10_ = 15.40, P = 0.0028). On fine sandy loam, the greater the work the greater the mass of the dislodged product (Figure 11c); the slope was positive and significantly different from zero (F_1,5_ = 106.9, P = 0.0001).

This predictable relationship did not hold for work done on the more resistant clayey soil; in that case, no significant relationship was found between work done and soil dislodged (F1,5 = 0.633, P = 0.463). The sandy loam and clayey soil slopes were significantly different from each other (F_1,10_ = 13.50, P = 0.0043).

The equation of the line for sandy loam in Figure 11c was used to calculate that the work needed to loosen material for a single average-sized bolus was 2.07 mJ. Because an adult spider would need to loosen 918 such boluses to excavate an average-sized burrow, the total external work done by a spider in loosening the sandy loam would be 1.90 J (2.07 mJ/bolus × 918 boluses; note conversion from mJ to J). For the equivalent calculation for clayey soil, in the absence of a significant relationship between work and product (Figure 11c), the mass of the average product (0.023 g) divided by the average work (4.146 mJ) was used to represent the product produced per unit of work. Based on that simplifying assumption, the total external work done by a spider in loosening one bolus (0.034 g) of the clayey soil was 5.63 J (6.13 mJ/bolus × 918 boluses; as above, note conversion from mJ to J), nearly three times the cost of loosening the same amount of sandy loam.

**Raising substrate to the surface.** The same 31.57 g of substrate that was loosened from the average burrow had to be transported to the surface during 918 vertical trips. At first, as the spider was just starting the excavation these trips were trivially short, measurable in millimeters, but as the burrow became deeper each trip became longer. The average trip to the surface had to be more than half the final depth of the burrow because most finished burrows are narrower at the surface and broader near the bottom (Figure 1), and some have a conspicuously enlarged chamber at the bottom. Based on the estimate that half of the mass of excavated material came from deeper than the top 60% of the final depth (13.16 cm) of the average burrow (Figure 1), the average mass-biased depth of the burrow was taken to be 7.9 cm.

Work against gravity is the product of the mass being moved upward, the vertical distance moved, and the acceleration of gravity. In the present case, for each trip the mass was the sum of the spider's mass (0.144 ± 0.041 g) and the average pellet mass (0.0344 g), the average distance was 0.0789 m, and as always gravity was 9.81 m/s^2^. The product, 0.138 mJ, was the external work done by the spider in one average trip to the surface carrying a bolus of substrate. The total external work was the product of this and 918, the number of such vertical trips: 0.127 J (after conversion from mJ to J).

**Dispersing the boluses of substrate.** When a spider throws a bolus of substrate, it uses its forelegs to accelerate the bolus. The work done during this acceleration gives the bolus its kinetic energy, calculated as:
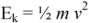

where *m* is the mass that was accelerated to velocity *v*. The kinetic energy of the bolus just after the spider released it was an accurate measure of the work used to propel the bolus. Frame-by-frame analysis of 10 bolus trajectories revealed velocities of 0.939 ± 0.119 m/s and, using the mean mass of a bolus as 0.0344 g, gave an average work per ejected bolus of 1.52 × 10^-5^ J. Multiplying that by 918 boluses gave 0.0139 J as the total cost of ejecting the substrate during burrow excavation.

Table 1 summarizes the energetic calculations described above for loosening the substrate, transporting it to the surface, and flinging it away from the burrow entrance.

## Discussion

### Meaning of the energetic costs

**Table 1.**
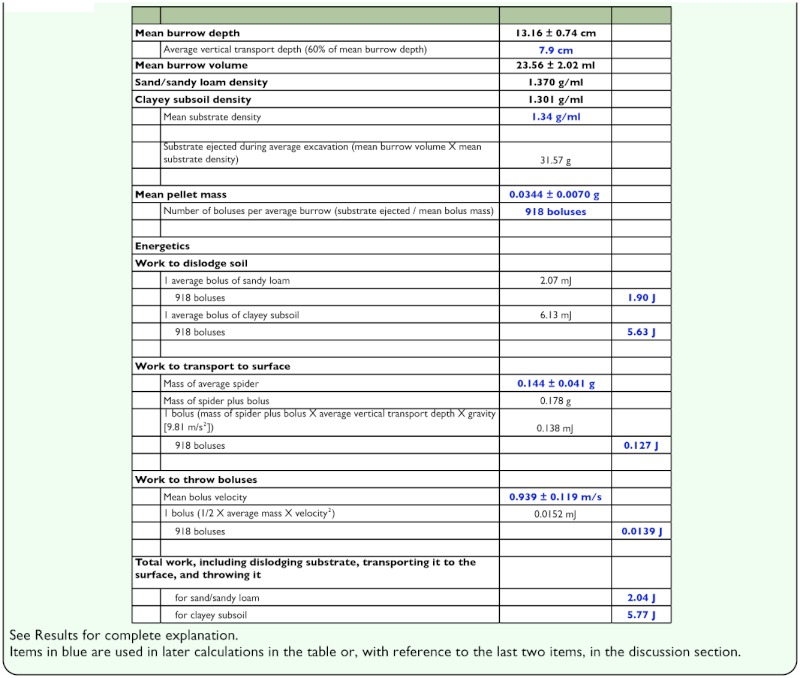
Parameters and values included in the calculation of the external work done by *Geolycosa* spp. during burrow construction.

The external work done by the spider while excavating its burrow included the work of loosening the sandy loam (1.9 J) or clayey soil (5.6 J), making multiple trips to the surface carrying boluses of substrate and the spider itself (0.13 J), and throwing the boluses away from the burrow entrance (0.014 J). The total of these costs, approximately 2.0 J in sandy loam or 5.8 J in clayey soil, constituted only the external work done by the spider; the costs did not include the additional physiological costs (the metabolic energy expended but lost as heat) incurred while doing the external work. Thus the total physiological cost of doing external work was the sum of the external work and the energy lost as heat.

The above distinction refers to muscle efficiency, the ratio of external work to total metabolic energy expenditure which can vary from 0 to 1 and which, under experimental conditions, usually lies between 0.20 to 0.25 (Pennycuick 1992; but see Humphreys 1978b for impact of the thermal environment for burrowing lycosids). This means that the total physiological cost of burrow construction for *Geolycosa* probably lies between 4 and 5× the measured external work: for sandy loam, 8–10 J; for clayey soil, 23–29 J.

How important to the spiders are these costs of burrow construction? Because the measures are conceptually so different, one cannot weigh energetic costs against stochastic benefits such as the avoidance of predation nor can one add known energetic costs to stochastic burrow-related costs such as the reduction in mate-attracting ability. It is possible, however, to look at excavation costs in comparison to the cost of producing an egg. This is a useful juxtaposition because the energy in a clutch of eggs is a reasonable and often-adopted index of reproductive effort, and reproductive effort, is a core component of fitness (Hirshfield and Tinkle 1975). A single egg of *Geolycosa* contains about 10.3 J (based on Marshall and Gittleman's report [1994] that an egg of *G. xera* has a wet mass of 1.33 mg, and on Anderson's estimation [1978] that spider eggs have an energy density of 27.3 J/mg ash-free dry mass, with ash-free dry mass being about 28.3 % of wet mass); so a *G. missouriensis* after constructing a burrow in sand/sandy loam has lost the energetic equivalent of a little less than one egg, and a *G. fatifera* after digging a burrow in clayey subsoil has lost the energetic equivalent of 2–3 eggs.

Complicating this analysis are two other considerations. First, these spiders often spend most of their lives in one burrow, enlarging it as they grow, so that the costs of construction are sometimes spread over the lifespan. On the other hand, a spider displaced by a predation attempt (Figure 2) or transport to a laboratory, readily constructed a new burrow, and if the displacement and new construction happened when the spider was an adult its lifetime constructions costs in effect would have doubled. Little is known about the frequency/probability of natural displacements, but that these parameters vary with species and habitat is clear (McQueen 1978, 1983; Miller 1989; Miller and Miller 1991; Marshall 1995). Second, the range of clutch sizes in *Geolycosa*, though known for only a few species, is very broad: *G. xera*, 24 (Marshall 1995); *G. fatifera*, 118 (Nicholas et al.); *G. missouriensis*, 133 and 179 (Richardson 1990; Nicholas et al.); *G. domifex*, 203 (McQueen 1978). Thus any definitive generalization about the proportion of an individual's lifetime reproductive effort that is lost due to burrow construction (e.g. 8.3% [2/24 eggs] vs. 1.0% [2/203 eggs]) is unwarranted.

On the other hand, calculations based just on *G. missouriensis* are instructive: these spiders burrow into sand/sandy loam that costs the equivalent of one egg per excavated burrow and have a mean clutch size of about 156 eggs (Richardson 1990; Nicholas et al.). If a female produces just one clutch in her lifetime and has had to excavate a full-sized burrow twice, her fecundity loss due to excavation costs is about 1.3% (2 eggs lost, 156 produced); if she produces two clutches in her lifetime and only had to excavate one burrow, her fecundity loss due to burrow construction costs is about 0.3% (1 egg lost, 312 produced). These are small percentages and compared to protection from predation, desiccation, and thermal instability (references in Introduction) may in that context be relatively unimportant.

Variation in excavation costs may, however, be substantial enough to play a part in natural selection. In Florida, *G. xera archboldi* digs burrows in loose sand (Marshall 1995), and in Michigan *G. wrightii* makes its burrows in lakeshore dunes (Richardson 1990). And both species have uncharacteristically high rates of burrow abandonment and reestablishment when compared to other *Geolycosa* species (Marshall 1995). These associations of low excavation costs and high relocation rates may indicate that, whatever the precipitating stimuli, the threshold for burrow abandonment is lower because the costs of new excavation are lower.

### Burrow depth vs. soil quality

Animals are known to optimize their behaviors and energy expenditures; for example, adjusting territory size in response to resource richness (e.g. in hummingbirds: Kodric-Brown and Brown 1978) or adjusting web position in response to predator risks and foraging rewards (e.g. in colonial webbuilding spiders: Rayor and Uetz 1993). The data presented in Figure 10 show that *G. fatifera*, the species found in burrows in the difficult-to-loosen clayey subsoil, dug significantly shallower burrows than did *G. missouriensis* when excavating burrows in the more easily dislodged sand/sandy loam. This may be an example of the same kind of optimization, or it could be a species difference unrelated to real-time (as opposed to evolutionary) optimization. A relatively simple reciprocal transplantation experiment will help to clarify the causes of the burrow depth difference.
